# *PSEN1* p.Thr116Ile Variant in Two Korean Families with Young Onset Alzheimer’s Disease

**DOI:** 10.3390/ijms19092604

**Published:** 2018-09-02

**Authors:** Eva Bagyinszky, Hye-Mi Lee, Vo Van Giau, Seong-Beom Koh, Jee Hyang Jeong, Seong Soo A. An, SangYun Kim

**Affiliations:** 1 Department of Bionano Technology, Gachon University, Sungnam 13120, Korea; navigator120@gmail.com (E.B.); giauvvo@gmail.com (V.V.G.); 2 Department of Neurology, Korea University Guro Hosipital, Korea University, Seoul 08308, Korea; platina84@naver.com (H.-M.L.); seongbeom.koh@gmail.com (S.-B.K.); 3 Department of Neurology, Ewha Womans University Mokdong Hospital, Ewha Womans University, Seoul 07985, Korea; jjeong@ewha.ac.kr; 4 Department of Neurology, Seoul National University College of Medicine & Neurocognitive Behavior Center, Seoul National University Bundang Hospital, Sungnam 13620, Korea

**Keywords:** young onset Alzheimer’s dementia, familial, presenilin-1, mutation, *PSEN1* Thr116Ile mutation

## Abstract

An in depth study of *PSEN1* mutation p.Thr116Ile (c.335C>T) is presented from two Korean families with autosomal dominant inheritance. Clinical manifestation of our patients included memory loss, attention deficits, visuospatial dysfunction, agnosia, aphasia, apraxia, and personality changes, which occurred in their 30s. *PSEN1* Thr116Ile was initially discovered in an Italian patient and two French families with early onset Alzheimer’s disease (EOAD) with similar age of onset. To verify the possible pathogenic mechanisms of mutation, *in silico* predictions and 3D modeling were performed. Structure predictions revealed significant aberrations in first hydrophilic loop (HL-I loop). The hydrophobic isoleucine could alter the loop orientation through increased hydrophobic contacts with the surrounding amino acids. Mutation could destroy a possible hydrogen bond between tyrosine 115 and threonine 116, which may affect the loop conformation. HL-I was confirmed as a conservative region of *PSEN1*, which may be critical in *PSEN1* functions. An additional pathogenic mutation, *PSEN1* Thr116Asn, was also found for the same residue, where the patient presented young onset AD (YOND). Other mutations in HL-I loop, such as Tyr115His and Glu120Asp, were described in patients with YOND, supporting the critical role of HL-I loop in *PSEN1* activity.

## 1. Introduction

Minority (5–15%) of all Alzheimer’s disease (AD) cases could occur under 65 years old, called early onset AD (EOAD), and even fewer AD patients are reported below 45 years old, called young onset AD (YOAD). Three genes were identified to be involved in EOAD/YOAD: amyloid prescursonr protein (*APP*, OMIM: 104760) on chromosome 21, presenilin 1 (*PSEN1*, OMIM: 104311) on chromosome 14, and presenlin 2 (*PSEN2*, OMIM: 600759) on chromosome 1. APP is a 770-amino acid-long protein, which contains the cleavage sites of γ and β secretase enzymes. PSEN1 and PSEN2 proteins are catalytic components of γ secretase complex, and function as asparatyl proteases. They could play critical role the processing APP and the production of amyloid beta (Aβ) [1,2]. Beside amyloid progression, additional possible factors may also play a significant role AD progression, for example immune deficiency or dysfunctions in autophagy system [1,3]. Most of these mutations in the above three genes presented an autosomal dominant inheritance pattern [1]. Interestingly, an autosomal recessive mutation, Ala673Val in *APP* gene, was also observed [4].

More than 200 mutations have been found in *PSEN1* (http://www.alzforum.org/mutations). Majority of patients with *PSEN1* mutations developed AD in their 40s and 50s. In addition, limited numbers of *PSEN1* mutations were reported in younger AD patients below 40s or even under 30 years of age [1,5,6,7,8]. Presenilins are parts of γ secretase complex, playing crucial roles in different neurological processes, such as synapse and memory formation and survival of neurons. Majority of *PSEN1* mutations were associated with gain-of function pathogenic mechanisms [9], which could enhance the production of longer amyloid fragments, such as Aβ42, Aβ43 or even Aβ48 [10]. Loss of *PSEN1* function may also result in enhanced disease progression by reducing the activity of α-secretase and the assembly of short amyloid peptide (Aβ40). Hence, increased and decreased productions of Aβ42 and Aβ40, respectively, would result in increased ratio of Aβ42/Aβ40. In addition, decreased Aβ40 levels in comparison to Aβ42 may be associated with the different degree of Aβ42 clearance, resulting an accumulation [9].

In this study, a *PSEN1* Thr116Ile (c.335C>T) mutation was discovered in two Korean YOAD families for the first time in Asia. Next, bioinformatic analyses and *in silico* protein structural predictions were also performed for the mutation. *PSEN1* Thr116Ile was previously reported in YOAD patients from Italy and France with similar age of onset in their 30s and 40s. All affected family members in the previous report and the current study developed disease symptoms with similar age of disease onset. Here, the clinical phenotypes of Korean patients are discussed in detail in comparison with the YOAD patients from Spain and France.

## 2. Results

### 2.1. Genetic Analysis

**Figure 1:**
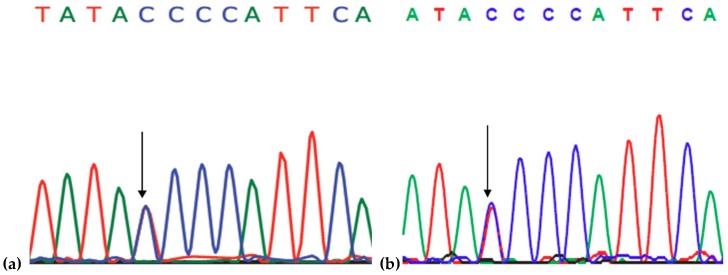

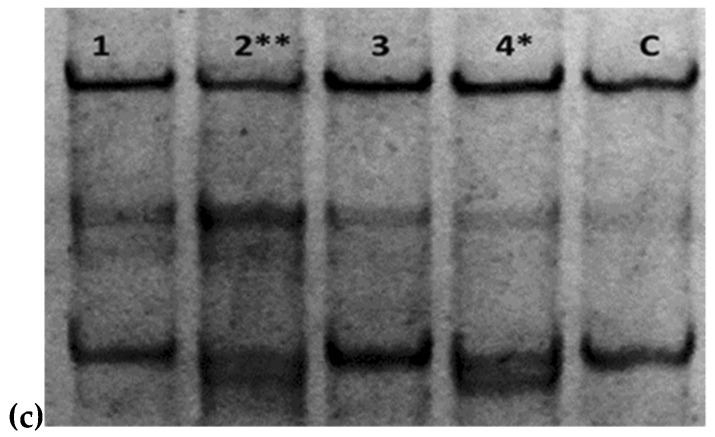
(**a**) Sequencing data of proband patient from Family 1 with *PSEN1* T116I; (**b**) sequencing data of proband patient from Family 1 with *PSEN1* T116I; and (**c**) SSCP data on *PSEN1* T116I in Family 1. Number 2** is the proband patient and Number 4* is her affected sister. Numbers 1 and 3 are the asymptomatic and unaffected sisters. The ”C” means the control band, a PCR product of an individual, who was verified as wild type for *PSEN1* exon 5. Star means that these individuals are affected with mutation.

Sequencing showed a heterozygous C->T transition at codon 116 (g.73640282C>T; c.335C>T), leading to a threonine (ACC) to isoleucine (ATC) exchange for codon 116 (Figure 1a,b) of *PSEN1*. No additional pathogenic mutations were observed in *APP* or *PSEN2* genes. SSCP also confirmed the presence of mutation (Figure 1c) from the different migration patterns between the mutation and wild type strains. *PSEN1* Thr116Ile was missing ExAC and 1000Genomes databases. *PSEN1* Thr116Ile was screened in the KRGDB database and was not present in healthy Koreans.

Three sisters of patient from Family 1 agreed to the genetic test, and one of her sisters (37 years of age at the time of genetic test) was positive for the mutation. The two other sisters (40 and 42 years at the time of genetic testing) did not show any phenotype of memory impairment, and they were negative for the mutation (Figure 1c).

### 2.2. In Silico Predictions and 3D Modeling

**Figure 2:**
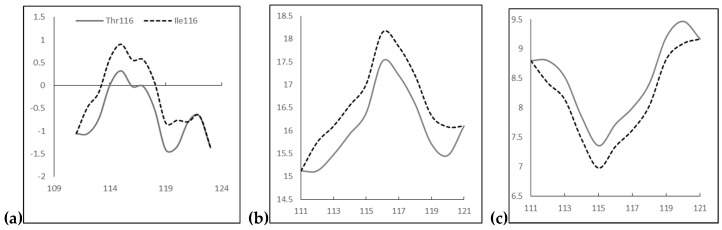
ExPASY prediction on *PSEN* T116I: (**a**) Kyte and Dootile hydrophobicity scores; (**b**) bulkiness scores; and (**c**) polarity scores.

PolyPhen2 revealed *PSEN1* Thr116Ile as probably damaging mutation in both HumDiv and HumVar scores, 1 and 0.999, respectively. Multiple sequence alignment confirmed that Thr116 was a conserved residue in the PSEN-like protein sequences among vertebrates. However, SIFT suggested this mutation as “tolerated” mutation with the scores of 0.07. PROVEAN scores revealed the mutation as deleterious with the scores of −5.462. ExPASY prediction suggested significant changes in hydrophobicity, bulkiness and polarity (Figure 2). Kyte and Doolittle hydrophobicity scores were increased significantly due to the mutation, from −0.022 (Thr116) to 0.556 (Ile116, Figure 2a). Bulkiness scores were also higher, changing from 17.513 (Thr116) to 18.139 (Ile116, Figure 2b). Polarity scores were decreased, from 7.722 (Thr116) to 7.344 (Ile116, Figure 2c). Changes in these parameters also affected the neighboring residues from residue 112 to residue 120.

**Figure 3:**
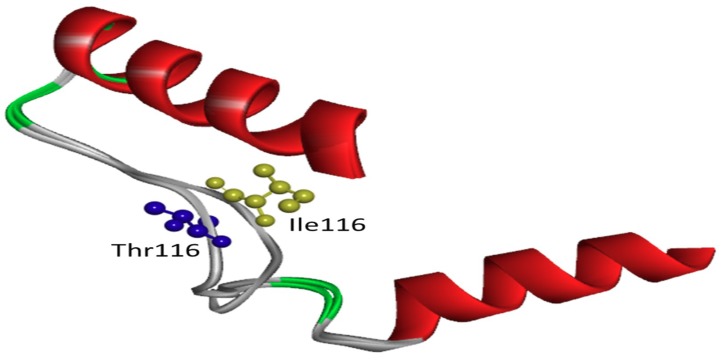
3D modeling on *PSEN1* Thr116Ile mutation, compared to the normal *PSEN1.* Threonine is labeled with blue while Isoleucine is labeled with yellow. Mutation could disturb significantly the HL-I loop structure.

**Figure 4:**
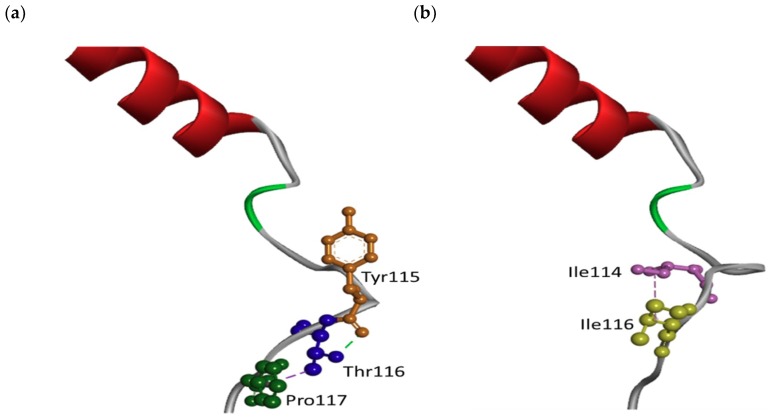
3D model on *PSEN1* Thr116Ile mutation, in terms intramolecular interactions. (**a**) Thr116 (blue) could form hydrogen bond with Tyr115 (orange) and may form hydrophobic interactions with Pro117 (green). (**b**) In the case of Ile116 (yellow), both contacts could be lost, and an additional hydrophobic interaction would be formed with Ile114 (purple).

The *in silico* 3D model on Thr116Ile revealed changes in the conformation of HL-I loop, which is known as a conservative region in *PSEN1* (Figure 3). Orientation of threonine and isoleucine were also different. The altered conformation of HL-I may also affect the conformation of TM-II domain of *PSEN1*. Threonine is a hydrophilic amino acid, which is able to form hydrogen bond, since it has a hydroxy group, and the missing hydrogen bond may result in intermolecular changes inside the loop. *In silico* predictions revealed that the normal Thr116 could form hydrogen bound with Tyr115 and might be contacted by hydrophobic interactions with Pro117 (Figure 4a). In the case of Ile116, both contacts could disappear, and an additional hydrophobic interaction would be formed with Ile114 (Figure 4b).

## 3. Discussion

In this study, a mutation (p.Thr116Ile) in *PSEN1* was presented as cause of EOAD in two Korean families, which had not been described among Asian patients previously. The substitution from the hydrophilic threonine residue to the hydrophobic isoleucine residue might result in aberrations in the *PSEN1* conformation. Both Korean patients with Thr116Ile presented strong family history of disease, since several ancestors and relatives were affected with the disorder. In both patients, the disease progression started in their late 30s or early 40s, and mild reduction could be seen in their MMSE scores (Table 1). MRI of patient from Family 1 revealed atrophy in the right temporal and parietal regions, and some vascular abnormality in the left frontal area. No significant atrophy was detected in patient from Family 2, but mild bitemporal hypometabolism was observed in FDG-PET (Table 1). In terms clinical symptoms, memory loss, visuospatial dysfunction and various neuropsychiatric symptoms, personality changes and speech disturbances were found in proband patient from Family 1. In proband patient of Family 2, memory loss appeared at the age of 37 [11], with altered mood and marked ideomotor apraxia.

*PSEN1* Thr116Ile was described initially by LaBella et al. (2004) in an early onset AD patient. The age of onset was 45 years, starting with forgetfulness, followed by memory loss, confusion and disorientation. Three-year history of progressive dementia could be seen in the patient. The exact family history remained unclear, since her grandmother and mother both died in their early 40s, but neither of their cause of death was associated with dementia (cancer and accident, respectively). Since the mutation was missing in 100 healthy non-related controls, it was suggested as a pathogenic mutation [12]. The second case of this mutation was reported by Raux et al. (2005) in four AD patients from the same family. Inheritance pattern was autosomal dominant, and the age of onset ranged 40–47 years. The NINCDS-ADRDA criteria confirmed that these patients had AD, but post-mortem analyses could not be performed [13]. A second French family was described by Wallon et al. (2012) in a large French study, where disease onset was 38–44 years of age. Disease duration could range 3–5 years. No detailed clinical data were mentioned [14]. Similar to the previously described disease cases from Europe, the affected members from the two Korean families developed disease between 38 and 47 years of age. Rapidly progressive dementia appeared in these patients, and they died before 50 years of age.

An additional mutation was found for the same residue, Thr116Asn (Table 2), reported in an EOAD family in Denmark. Autopsy confirmed that neuropathology was consistent with AD diagnosis. Age of onset ranged 38–41 years. This mutation was associated with rapid progression of disease, since the duration from the first clinical symptoms to the death ranged 4–8 years. Earlier onset of disease (30–33 years) was observed in a French family, but there was no information on the clinical symptoms in this family [15].

**Figure 5:**
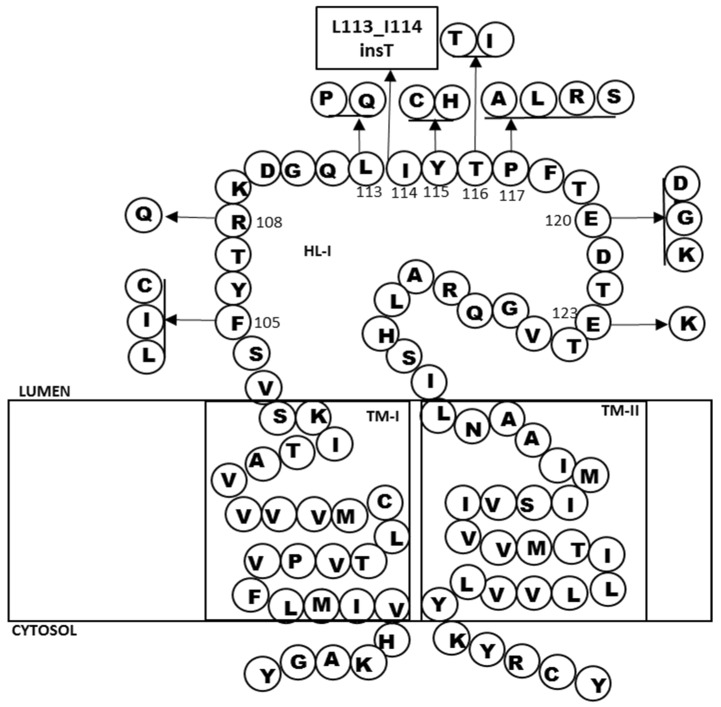
Mutations, located in the HL-I of PSEN1 protein.

The hydrophilic loop (HL)-I is located between transmembrane (TM)-I and TM-II of *PSEN1*, and 18 mutations were observed in this region (Table 3 and Figure 5). The majority of them (19) were missense mutations. In addition, one splice site mutation was also reported, resulting in an insertion (Leu113_Ile114insT). Patients with these mutations usually developed AD, but one mutation, *PSEN1* Leu113Pro, was associated with FTD [16]. Pathogenic nature of mutation Arg108Gln was refuted, since it co-existed with a known mutation in *APP* [17]. AD patients could present additional phenotypes too, such as Parkinsonsim (Pro105Leu) [18], myoclonic jerks (Arg108Gln, Leu113Pro, Tyr115His, Pro117Leu, Pro117Ser, snd Pro117Arg) [17,19,20,21,22], seizures (Leu113Gln, Leu113Pro, Tyr115His, and Pro117Leu) [17,18,19,20], language–behavioral impairments (such as Tyr105Cys and Glu120Asp) [21,22], ataxia (Pro117Ala) [21], or spastic paraparesis (Glu120Gly) [22]. Age of disease onset was usually under 60 years of age. In addition, several patients with some of these mutations (Leu113Pro, Tyr115Cys, Pro117Leu, Pro117Ser, and Glu120Gly) developed young onset AD under 40 years of age [17,18,19,22,23]. The majority of these residues were conserved, but *PSEN1* Glu123Lys seemed to be not conserved in *PSEN2* [24]. These mutations may prove that HL-I could be an important region in *PSEN1*. This loop is located in the lumen, and was found to be a highly conserved region in *PSEN1* [12]. Gong et al. (2010) performed generated mutations in HL-I and TM-II regions of *PSEN1*, and confirmed that importance of these regions in γ secretase activity. These domains may not be involved in γ secretase complex formation, and in the recruitment to other components (APP, Notch or N-cadherin) to PSEN1 protein. Instead, they might play a critical role in the endoproteolysis and catalysis of γ secretase substrates. In addition, HL-I and TM-II might be important in regulating the docked substrates to the enzyme [25]. Additional studies by Tagaki-Niidome et al. (2015) suggested that HL-I and the C-terminal may be important in the γ and ε cleavage. These two regions may play a critical role in substrate binding site in γ secretase complex [26,27].

Limitations of our study are that no in vitro studies could be performed to confirm how *PSEN1* T116Ile could be involved in disease progression, since no CSF could be obtained in the patients, and biomarkers (Aβ, total Tau and phospho-Tau) could not be measured. In addition, since several of them refused the genetic test, segregation could not be proven even though inheritance seemed to be autosomal dominant in both families.

## 4. Materials and Methods

### 4.1. AD Patients and Their Families

#### 4.1.1. Family 1

**Figure 6:**
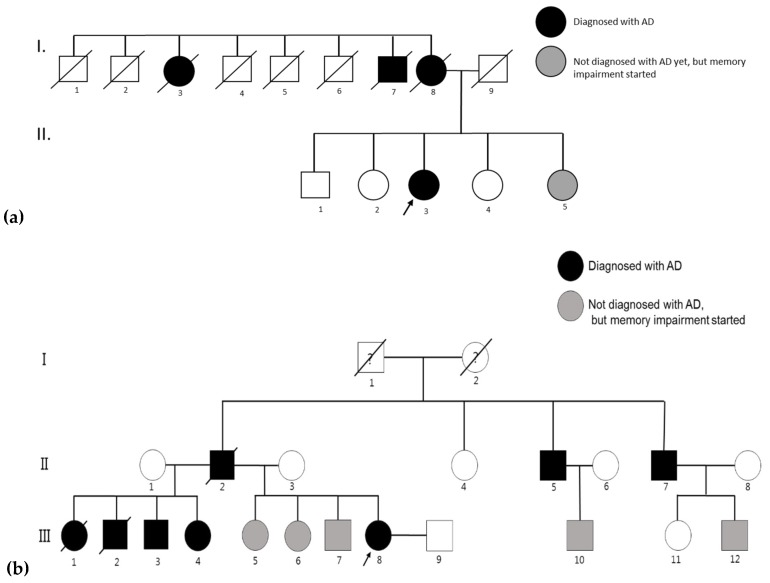
(**a**) Family tree of patient with *PSEN1* T116I mutation (Family 1); and (**b**) family tree of patient with *PSEN1* T116I mutation (Family 2). This figure was adapted and reprinted with the permission from Dove Medical Press (Clinical Interventions in Aging) [11]. White circles mean asymptomatic patients, which were not diagnosed with disease. Family members which were crossed out, already died. Arrows show the proband patient. Question mark at the grandparents means that their disease status is unclear.

A 41-year-old woman (II-3, Figure 6a) visited the Korea University Guro Hospital with gradually impaired cognitive function over the previous three years. The first symptoms appeared at the age of 38 years when mild forgetfulness was described by her husband. She had managed a travel agency, but she dissolved the company because of impairment of episodic memory and poor concentration. Her memory problems became prominent progressively with personality changes. She denied any social history of substance abuse or toxin exposure. Her past medical history was otherwise negative for medical illnesses. Her family history revealed that her mother (I-8) died at the age of 44 without genetic analysis, after experiencing seven years of memory impairment, visuospatial dysfunction and various neuropsychiatric symptoms. In addition, her maternal aunt (I-3) and maternal uncle (I-7) also experienced severe cognitive impairment from their mid-30s. The proband patient was the third of five children, and her younger sister (II-5) also experienced memory problems without impairment of activities of daily living recently. The three sisters (II-2, II-4 and II-5) of patient agreed to undergo a screen for mutation, however, her currently asymptomatic brother (II-1) declined the genetic test or to provide any information on himself.

**Figure 7:**
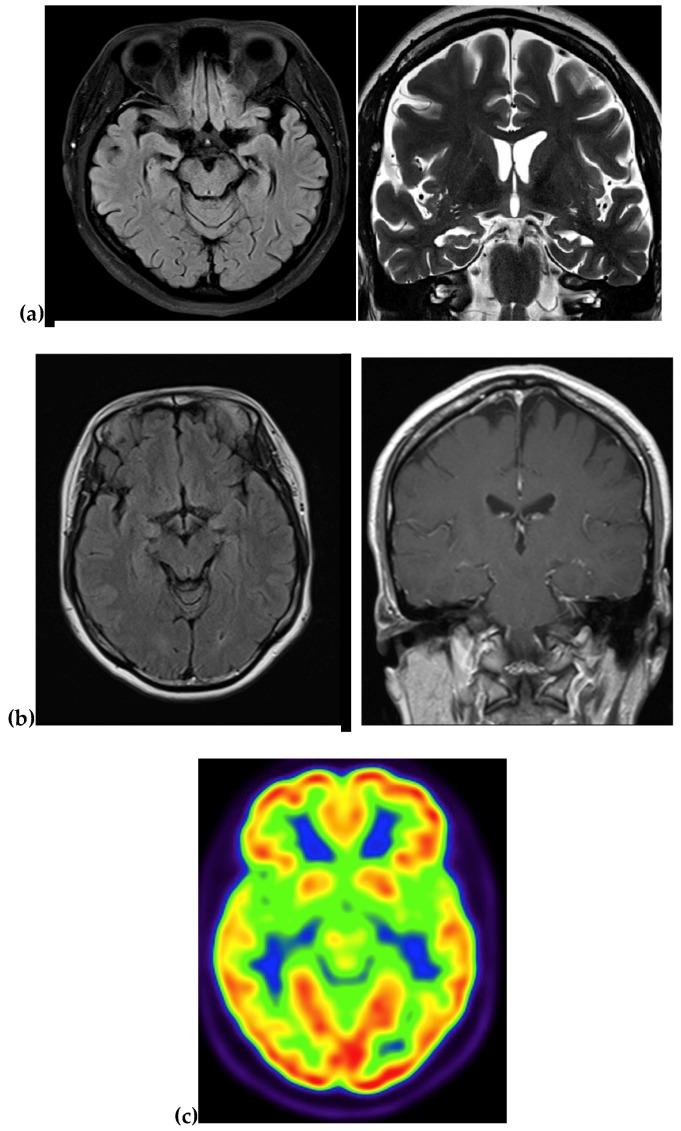
(**a**) MRI data of proband patient of Family 1. Atrophy could be seen in the right temporal and parietal regions. A vascular abnormality also appeared in the left frontal area. (**b**) MRI data of proband patient of Family 2. (**c**) PET data from Family 2.

Upon neurological examination, she showed slurred speech, mild ideomotor apraxia and impairment of short-term and long-term memory functions. Her Korean version of the mini-mental state examination (K-MMSE) score was 24/30, and global deterioration scale score was 3 (mild cognitive decline). Magnetic resonance imaging (MRI) of the brain revealed mild diffuse cortical atrophy. In the imaging data, atrophy could be seen in the right temporal and parietal regions. Vascular abnormalities also appeared in the left frontal area (Figure 7a). Laboratory screening was negative for vasculitic, metabolic, or infectious causes. Her symptoms gradually progressed, and there was no response to acetylcholine esterase inhibitor. After two years, her K-MMSE score was 18/30 and global deterioration scale score was 4 (moderate cognitive decline). At the age of 44 years, she became wheelchair-bound, and she was put in a dementia hospital. Blood samples for the genetic testing were collected from the proband patient. Subsequent DNA sequencings of her three sisters were also performed, and *PSEN1* Thr116Ile mutation was found in her younger sister (II-5), and she already developed mild memory impairment. The currently asymptomatic sisters (II-2 and II-4) were negative for the mutation. After the genetic diagnosis of YOAD, she was monitored regularly.

#### 4.1.2. Family 2

Proband patient was part of Clinical Research Center for Dementia of South Korea (CREDOS) project, which analyzed *APP*, *PSEN1* and *PSEN2* mutations in 100 EOAD patients. Clinical details and scientific predictions were not included in the previously reported study [11]. In this manuscript, a detailed description of this case with detailed clinical, imaging data, and structure prediction analyses are presented. A 43-year-old woman (II-8, Figure 6b) visited Ewha Woman’s University Mokdong Hospital with the presentation of gradual cognitive dysfunction, which started at 36 years of age. Her first symptoms were mild forgetfulness, repeating same conversations and forgetting valuable objects. Recently, her memory impairment became worse, she could not remember when she had meal, eating several times a day, and who she had met or where she had been. Her mood also changed to apathetic because of her impairment from previous concern or worrying about herself. She was still able to manage household, taking care of her children, but the cleanliness diminished. She was a nutritionist at high school, but retired the year before the first visit. At neurological examination, she showed brisk deep tendon reflexes, especially in knee jerk and mild paratonia. Marked ideomotor apraxia was observed. Her Korean version of the mini-mental state examination (K-MMSE) score was 24/30, clinical dementia rating scale was 0.5 (sum of boxes 2.5) and global deterioration scale score was 3 (mild cognitive decline), but showed moderate impairment of short-term and long-term memory function in detailed neuropsychological test. Her past medical history was negative for any medical illness, substance abuse or toxin exposure. Her family presented strong family history of dementia. No information was available on her grandparents, but his father (II-2) died at the age of 65, after experiencing 10 years of dementia. In addition, her paternal uncles (II-5 and II-7) also suffered from dementia from their mid-forties. Proband patient was the last of eight children from the two marriages of II-2. All of her older half-sisters and half-brothers (III-1, III-2, III-3, and III-4) suffered from dementia. Her siblings (III-5, III-6, and III-7) and her cousins (III-10 and III-12) also developed mild memory problems. All living family members refused the genetic test. Blood samples for genetic testing were collected from the proband patient.

Magnetic resonance imaging (MRI) of the brain revealed no atrophy or vascular ischemic lesion (Figure 7b), but mild bitemporal hypometabolism in 18F fluorodeoxyglucose—Positron Emission Tomography (FDG-PET, Figure 7c) was suggested. Laboratory screening was negative for vasculitic, metabolic, or infectious causes. After three years, her symptoms progressed to more memory loss, ADL impairment, visual hallucination and dressing apraxia. Her physical activity was still intact, being able to walk and do simple household tasks. Her K-MMSE score declined to 19/30, clinical dementia rating scale of 1 (sum of boxes 8) and global deterioration scale score was 5 (moderate cognitive decline).

### 4.2. Genetic Analysis

The study was approved by the institutional review boards of the Korea University Guro Hospital and Ewha Woman’s University Mokdong Hospital (13-21A-06; 03 July 2013). Written informed consents were obtained from all patients (or their caregivers) who participated in the study.

Blood samples of patients were centrifuged at 800× *g* for a 30 min, followed by the isolation of white blood cells (Buffy coat). DNA was purified by GeneAll blood kit (Seoul, Republic of Korea), and kept at −20 °C. Standard sequencing was performed for *APP* exon 16 and 17 [35,36], and for the coding region (exon 3–12) of *PSEN1* and *PSEN2* [21,37] genes. In addition, the coding regions of *PRNP* [38], *PGRN* [39] and *MAPT* [40] genes were also screened for pathogenic mutations. Before sequencing, polymerase chain reaction (PCR) products were purified by Expin PCR kit (Seoul, Republic of Korea). Sequencing was performed by BioNeer Inc. company (Dajeon, Republic of Korea). BigDye Terminator Cycle Sequencing kit (Seoul, Republic of Korea) was used for the sequencing reactions and ABI 3730XL DNA Analyzer was used to screen the chromatogramms (Bioneer Inc., Dajeon, Korea). Sequences were aligned by NCBI BLAST (http://blast.ncbi.nlm.nih.gov/Blast.cgi) and screened with DNA Baser software (http://www.dnabaser.com). Mutations and sequence mutations were identified and compared against the NCBI Gene (http://www.ncbi.nlm.nih.gov/gene) and UniProt (http://www.uniprot.org) databases. Mutations were screened in Korean Reference Genome Database (KRGDB, http://152.99.75.168/KRGDB/menuPages/intro.jsp), where 622 asymptomatic Korean individuals were screened by whole genome sequencing. Mutations were also checked in larger databases, such as 1000Genomes (http://www.internationalgenome.org/) and Exome Aggregation Consortium (ExAC, http://exac.broadinstitute.org/).

### 4.3. Single Strand Conformation Polymorphism (SSCP)

PCR-SSCP was based on the different mobility of single stranded DNA in gel from the altered conformations due to the mutations in comparison with the wild type [41]. Formamide loading buffer was added to the PCR products (50:50), and incubated at 98 °C for 10 min, followed by cooling down on ice for 10 min. Native 12% polyacrylamide gel electrophoresis (PAGE) was used for the analysis. The running time was 15–21 h, at 100 V, on ice with TBE running buffer. The differential bands were visualized by SYBR Gold staining by following the manufacturer’s protocol (Invitrogen Inc. Boston, MA, USA).

### 4.4. In Silico Screening and Structure Predictions

Possible pathogenic nature of mutations could be analyzed using simple online software tools, such as PolyPhen2 (http://genetics.bwh.harvard.edu/pph2) [42], Sorting intolerant to tolerant (SIFT, http://sift.jcvi.org/) [43] or Protein Variation Effect Analyzer (PROVEAN, http://provean.jcvi.org/index.php) [44]. ExPasy prediction was also performed based on different parameters, including hydrophobicity scores, bulkiness and polarity [45]. The 3D modeling of normal and mutant PSEN1 protein structures were performed by the Raptor X web server (http://raptorx.uchicago.edu/) [46]. This protein structure prediction server produced the 3D protein structures based on the homology modeling from the given protein sequences. The normal and mutant PSEN1 protein sequence was aligned by Discovery Studio 3.5 Visualizer tool (Accelrys, San Diego, CA, USA).

## 5. Conclusions

In conclusion, our report confirmed that *PSEN1* Thr116Ile mutation was causative of an autosomal dominant EOAD. *In silico* predictions were performed to estimate the possible role of mutation, and confirmed that it could disturb the HL-I loop, resulting in significant possible disturbances in γ secretase functions.

## Figures and Tables

**Table 1:** Comparison of EOAD cases, associated with *PSEN1* T116I mutation.

Case	First Case	Second Case	Third Case	Korean-1	Korean-2
**Country**	Italy	France	France	Korea	Korea
**Age of onset (years)**	45 years	40–47 years	38–44 years	41 years	38 years
**Disease**	EOAD	EOAD	EOAD	EOAD	EOAD
**Imaging**	Atrophy and fronto-parieto-temporal enlargement of cortical sulci	NA	NA	MRI: atrophy in the right temporal and parietal regions PET: NA	MRI: Atrophy in the medial region PET: reduced metabolism in temporal and parietal region
**MMSE**	14/30	NA	NA	Initially 24/30, later 18/30	23/30
**Clinical phenotype**	Memory loss, confusion and disorientation, followed by progressive memory loss	No detailed information, but patients fulfilled the NINCDS-ADRDA criteria	No detailed information	Memory impairment, confusion, visuospatial dysfunction, speech disturbances.	Impairment in memory and mood, marked ideomotor apraxia
**Family history**	Unknown	Familial	Probable familial	Familial	Familial
**References**	[12]	[13]	[14]	Our data	Our data [11]

**Table 2:** Comparison of *PSEN1* Thr116Ile with Thr116Asn.

Mutation	Thr116Asn	Thr116Ile
**Pathogenicity**	Pathogenic	Pathogenic
**Age of onset**	35–41 years (Familial)	40–47 years (Familial and de novo)
**Clinical phenotype**	EOAD (no clinical data)	EOAD
**PolyPhen2 scores (HumDiv)**	1.00 (probably damaging)	1.00 (probably damaging)
**SIFT scores**	0 (damaging)	0.07 (tolerated)
**References**	[12,13,14]	[15]

**Table 3:** Mutations, located in *PSEN1* HL-I.

Mutation	Dx	Clinical Symptoms/Pathology	Age of Onset	Family History	Functional Studies	References
Phe105Cys	AD	Memory impairment and behavioral changes	45–60 years	Positive	NA	[28]
Phe105Ile	AD	NA	53–58 years	Positive	NA	[19]
Ple105Leu	AD	Parkinson’s like symptoms, severe dementia	52 years	Probable positive	NA	[18]
Arg108Gln	AD	Progressive cognitive decline, myoclonic jerks	45 years	Segregation could not proven	NA	[17]
L113_I114insT	AD	Postmortem studies confirmed the AD	34–45 years	Familial	3.4-fold higher Aβ42 levels	[16]
Leu113Pro	FTD	Behavioral impairments, myoclonic jerks, seizures	38–50 years	Familial	NA	[19]
Leu113Gln	AD	Rapid progressive dementia, drop attacks, myoclonic seizures, and bilateral spasticity.	33–36 years	Familial	NA	[18]
Tyr115Cys	AD	Postmortem studies confirmed the AD	39–45 years	Familial	5.4-fold higher Aβ42 levels	[21]
Tyr115His	AD	Epileptic seizure, myoclonus	35–40 years	Familial	Increased levels of Aβ42, lower Aβ40	[29]
Thr116Ile	Was discussed in details in Table 1 and Table 2					
Thr116Asn						
Pro117Ala	AD	Issues with balance, tremor, ataxia	29–35 years	Familial	Elevated Aβ42/40 ratio	[23]
Pro117Leu	AD	Progressive memory impairment, mood swings, seizure, myoclonus	24–33 years	Familial/de novo	Elevated Aβ42, inhibited neural overgrowth	[20]
Pro117Arg	AD	Apathy, behavioral changes, seizures, myoclonus, gait impairment	33–36 years	Unknown/ familial	Elevated total Aβ	[30]
Pro117Ser	AD	Personality changes, severe dementia, seizures, myoclonus, tremor	29–33 years	Familial	Elevated Aβ42 levels	[31]
Glu120Asp	AD	Language impairment, seizures, disorientation	34–53 years	Familial	NA	[22]
Glu120Gly	AD	Memory and cognitive decline, seizure, gait disturbances	30–39 years	Familial	NA	[32]
Glu120Lys	AD	Spastic paraparesis	43–45 years	Familial/ unknown	Elevated Aβ42/40 ratio	[33,34]
Glu123Lys	AD	Progressive aphasia, reduced visuospatial activity	56–62 years	Familial	NA	[24]

## References

[1] Bagyinszky E., Youn Y.C., An S.S., Kim S.Y. (2014). The genetics of Alzheimer’s disease. Clin. Interv. Aging.

[2] Wolfe M.S. (2010). Structure, mechanism and inhibition of gamma-secretase and presenilin-like proteases. Biol. Chem..

[3] Azarnia Tehran D., Kuijpers M., Haucke V. (2018). Presynaptic endocytic factors in autophagy and neurodegeneration. Curr. Opin. Neurobiol..

[4] Di Fede G., Catania M., Morbin M., Rossi G., Suardi S., Mazzoleni G., Merlin M., Giovagnoli A.R., Prioni S., Erbetta A. (2009). A recessive mutation in the APP gene with dominant-negative effect on amyloidogenesis. Science.

[5] Zou Z., Liu C., Che C., Huang H. (2014). Clinical genetics of Alzheimer’s disease. Biomed. Res. Int..

[6] Ertekin-Taner N. (2007). Genetics of Alzheimer’s disease: A centennial review. Neurol. Clin..

[7] Campion D., Flaman J.M., Brice A., Hannequin D., Dubois B., Martin C., Moreau V., Charbonnier F., Didierjean O., Tardieu S. (1995). Mutations of the presenilin I gene in families with early-onset Alzheimer’s disease. Hum. Mol. Genet..

[8] Campion D., Dumanchin C., Hannequin D., Dubois B., Belliard S., Puel M., Thomas-Anterion C., Michon A., Martin C., Charbonnier F. (1999). Early-onset autosomal dominant Alzheimer disease: Prevalence, genetic heterogeneity, and mutation spectrum. Am. J. Hum. Genet..

[9] Xia D., Watanabe H., Wu B., Lee S.H., Li Y., Tsvetkov E., Bolshakov V.Y., Shen J., Kelleher R.J. (2015). Presenilin-1 knockin mice reveal loss-of-function mechanism for familial Alzheimer’s disease. Neuron.

[10] Chávez-Gutiérrez L., Bammens L., Benilova I., Vandersteen A., Benurwar M., Borgers M., Lismont S., Zhou L., Van Cleynenbreugel S., Esselmann H. (2012). The mechanism of γ-secretase dysfunction in familial Alzheimer disease. EMBO J..

[11] An S.S., Park S.A., Bagyinszky E., Bae S.O., Kim Y.J., Im J.Y., Park K.W., Park K.H., Kim E.J., Jeong J.H. (2016). A genetic screen of the mutations in the Korean patients with early-onset Alzheimer’s disease. Clin. Interv. Aging.

[12] La Bella V., Liguori M., Cittadella R., Settipani N., Piccoli T., Manna I., Quattrone A., Piccoli F. (2004). A novel mutation (Thr116Ile) in the presenilin 1 gene in a patient with early-onset Alzheimer’s disease. Eur. J. Neurol..

[13] Raux G., Guyant-Marechal L., Martin C., Bou J., Penet C., Brice A., Hannequin D., Frebourg T., Campion D. (2005). Molecular diagnosis of autosomal dominant early onset Alzheimer’s disease: An update. J. Med. Genet..

[14] Wallon D., Rousseau S., Rovelet-Lecrux A., Quillard-Muraine M., Guyant-Maréchal L., Martinaud O., Pariente J., Puel M., Rollin-Sillaire A., Pasquier F. (2012). The French series of autosomal dominant early onset Alzheimer’s disease cases: Mutation spectrum and cerebrospinal fluid biomarkers. J. Alzheimers Dis..

[15] Romero I., Jorgensen P., Bolwig G., Fraser P.E., Rogaeva E., Mann D., Havsager A.M., Jørgensen A.L. (1999). A presenilin-1 Thr116Asn substitution in a family with early-onset Alzheimer’s disease. Neuroreport.

[16] Tysoe C., Whittaker J., Xuereb J., Cairns N.J., Cruts M., Van Broeckhoven C., Wilcock G., Rubinsztein D.C. (1998). A presenilin-1 truncating mutation is present in two cases with autopsy-confirmed early-onset Alzheimer disease. Am. J. Hum. Genet..

[17] Dobricic V., Stefanova E., Jankovic M., Gurunlian N., Novakovic I., Hardy J., Kostic V., Guerreiro R. (2012). Genetic testing in familial and young-onset Alzheimer’s disease: Mutation spectrum in a serbian cohort. Neurobiol. Aging.

[18] Finckh U., Kuschel C., Anagnostouli M., Patsouris E., Pantes G.V., Gatzonis S., Kapaki E., Davaki P., Lamszus K., Stavrou D. (2005). Novel mutations and repeated findings of mutations in familial Alzheimer disease. Neurogenetics.

[19] Raux G., Gantier R., Thomas-Anterion C., Boulliat J., Verpillat P., Hannequin D., Brice A., Frebourg T., Campion D. (2000). Dementia with prominent frontotemporal features associated with L113P presenilin 1 mutation. Neurology.

[20] Wisniewski T., Dowjat W.K., Buxbaum J.D., Khorkova O., Efthimiopoulos S., Kulczycki J., Lojkowska W., Wegiel J., Wisniewski H.M., Frangione B. (2008). A novel Polish presenilin-1 mutation (P117L) is associated with familial Alzheimer’s disease and leads to death as early as the age of 28 years. Neuroreport.

[21] Cruts M., van Duijn C.M., Backhovens H., Van den Broeck M., Wehnert A., Serneels S., Sherrington R., Hutton M., Hardy J., St George-Hyslop P.H. (1998). Estimation of the genetic contribution of presenilin-1 and -2 mutations in a population-based study of presenile Alzheimer disease. Hum. Mol. Genet..

[22] Poorkaj P., Sharma V., Anderson L., Nemens E., Alonso M.E., Orr H., White J., Heston L., Bird T.D., Schellenberg G.D. (1998). Missense mutations in the chromosome 14 familial Alzheimer’s disease presenilin 1 gene. Hum. Mutat..

[23] Anheim M., Hannequin D., Boulay C., Martin C., Campion D., Tranchant C. (2007). Ataxic variant of Alzheimer’s disease caused by Pro117Ala *PSEN*1 mutation. J. Neurol. Neurosurg. Psychiatry.

[24] Yasuda M., Maeda K., Hashimoto M., Yamashita H., Ikejiri Y., Bird T.D., Tanaka C., Schellenberg G.D. (1999). A pedigree with a novel presenilin 1 mutation at a residue that is not conserved in presenilin 2. Arch. Neurol..

[25] Gong P., Vetrivel K.S., Nguyen P.D., Meckler X., Cheng H., Kounnas M.Z., Wagner S.L., Parent A.T., Thinakaran G. (2010). Mutation analysis of the presenilin 1 N-terminal domain reveals a broad spectrum of gamma-secretase activity toward amyloid precursor protein and other substrates. J. Biol. Chem..

[26] Winblad B., Kosik K., Haltia M., Poyhonen M., Dickson D., Mann D., Neary D., Snowdon J., Lantos P., Lannfelt L. (1995). The structure of the presenilin 1 (S182) gene and identification of six novel mutations in early onset AD families. Nat. Genet..

[27] Takagi-Niidome S., Sasaki T., Osawa S., Sato T., Morishima K., Cai T., Iwatsubo T., Tomita T. (2015). Cooperative roles of hydrophilic loop 1 and the C-terminus of presenilin 1 in the substrate-gating mechanism of γ-secretase. J. Neurosci..

[28] Jiao B., Tang B., Liu X., Xu J., Wang Y., Zhou L., Zhang F., Yan X., Zhou Y., Shen L. (2014). Mutational analysis in early-onset familial Alzheimer’s disease in Mainland China. Neurobiol. Aging.

[29] Campion D., Flaman J.M., Brice A., Hannequin D., Dubois B., Martin C., Moreau V., Charbonnier F., Didierjean O., Tardieu S. (1995). Mutations of the presenilin I gene in families with early-onset Alzheimer’s disease. Hum. Mol. Genet..

[30] Zekanowski C., Styczyńska M., Pepłońska B., Gabryelewicz T., Religa D., Ilkowski J., Kijanowska-Haładyna B., Kotapka-Minc S., Mikkelsen S., Pfeffer A. (2003). Mutations in presenilin 1, presenilin 2 and amyloid precursor protein genes in patients with early-onset Alzheimer’s disease in Poland. Exp. Neurol..

[31] Dowjat W.K., Wisniewski T., Efthimiopoulos S., Wisniewski H.M. (1999). Inhibition of neurite outgrowth by familial Alzheimer’s disease-linked presenilin-1 mutations. Neurosci. Lett..

[32] Lladó A., Sánchez-Valle R., Rey M.J., Mercadal P., Almenar C., López-Villegas D., Fortea J., Molinuevo J.L. (2010). New mutation in the PSEN1 (E120G) gene associated with early onset Alzheimer’s disease. Neurologia.

[33] Hutton M., Busfield F., Wragg M., Crook R., Perez-Tur J., Clark R.F., Prihar G., Talbot C., Phillips H., Wright K. (1996). Complete analysis of the presenilin 1 gene in early onset Alzheimer’s disease. Neuroreport.

[34] Ryan N.S., Nicholas J.M., Weston P.S.J., Liang Y., Lashley T., Guerreiro R., Adamson G., Kenny J., Beck J., Chavez-Gutierrez L. (2016). Clinical phenotype and genetic associations in autosomal dominant familial Alzheimer’s disease: A case series. Lancet. Neurol..

[35] Schellenberg G.D., Pericak-Vance M.A., Wijsman E.M., Moore D.K., Gaskell P.C., Yamaoka L.A., Bebout J.L., Anderson L., Welsh K.A., Clark C.M. (1991). Linkage analysis of familial Alzheimer disease, using chromosome 21 markers. Am. J. Hum. Genet..

[36] Tanzi R.E., Vaula G., Romano D.M., Mortilla M., Huang T.L., Tupler R.G., Wasco W., Hyman B.T., Haines J.L., Jenkins B.J. (1992). Assessment of amyloid β-protein precursor gene mutations in a large set of familial and sporadic alzheimer disease cases. Am. J. Hum. Genet..

[37] Kamimura K., Tanahashi H., Yamanaka H., Takahashi K., Asada T., Tabira T. (1998). Familial Alzheimer’s disease genes in Japanese. J. Neurol. Sci..

[38] Jeong B.H., Ju W.K., Huh K., Lee E.A., Choi I.S., Im J.H., Choi E.K., Kim Y.S. (1998). Molecular analysis of prion protein gene (PRNP) in Korean patients with Creutzfeldt-Jakob disease. J. Korean Med. Sci..

[39] Cruts M., Gijselinck I., van der Zee J., Engelborghs S., Wils H., Pirici D., Rademakers R., Vandenberghe R., Dermaut B., Martin J.J. (2006). Null mutations in progranulin cause ubiquitin-positive frontotemporal dementia linked to chromosome 17q21. Nature.

[40] Rizzu P., Van Swieten J.C., Joosse M., Hasegawa M., Stevens M., Tibben A., Niermeijer M.F., Hillebrand M., Ravid R., Oostra B.A. (1999). High prevalence of mutations in the microtubule-associated protein tau in a population study of frontotemporal dementia in the Netherlands. Am. J. Hum. Genet..

[41] Hayashi K. (1991). PCR-SSCP: A simple and sensitive method for detection of mutations in the genomic DNA. PCR Methods Appl..

[42] Adzhubei I.A., Schmidt S., Peshkin L., Ramensky V.E., Gerasimova A., Bork P., Kondrashov A.S., Sunyaev S.R. (2010). A method and server for predicting damaging missense mutations. Nat. Methods..

[43] Ng P.C., Henikoff S. (2003). SIFT: Predicting amino acid changes that affect protein function. Nucleic Acids Res..

[44] Choi Y., Sims G.E., Murphy S., Miller J.R., Chan A.P. (2012). Predicting the functional effect of amino acid substitutions and indels. PLoS ONE.

[45] Gasteiger E., Gattiker A., Hoogland C., Ivanyi I., Appel R.D., Bairoch A. (2003). ExPASy: The proteomics server for in-depth protein knowledge and analysis. Nucleic Acids Res..

[46] Källberg M., Wang H., Wang S., Peng J., Wang Z., Lu H., Xu J. (2012). Template-based protein structure modeling using the RaptorX web server. Nat. Protoc..

